# SARS-CoV-2 infection in general practice in Ireland: a seroprevalence study

**DOI:** 10.3399/BJGPO.2021.0038

**Published:** 2021-06-30

**Authors:** Michael E O'Callaghan, Elizabeth Ryan, Cathal Walsh, Peter Hayes, Monica Casey, Pat O'Dwyer, Aidan Culhane, John W Duncan, Pat Harrold, Jarlath Healy, Emmet Kerin, Eimear Kelly, Conor Hanrahan, Ger P Lane, Bernie Lynch, Paraic Meaney, Billy O'Connell, Jim Galvin, Norelee Kennedy, Paul Burke, Nuala H O'Connell, Colum P Dunne, Liam G Glynn

**Affiliations:** 1 School of Medicine, University of Limerick, Limerick, Ireland; 2 Department of Biological Sciences, University of Limerick, Limerick, Ireland; 3 Health Research Institute, University of Limerick, Limerick, Ireland; 4 Department of Mathematics and Statistics, University of Limerick, Limerick, Ireland; 5 School of Allied Health, University of Limerick, Limerick, Ireland; 6 University Hospital Limerick, Limerick, Ireland; 7 Health Research Board Primary Care Clinical Trials Network Ireland, Galway, Ireland

**Keywords:** general practice, point-of-care systems, COVID-19, SARS-CoV-2, seroepidemiologic studies, serologic tests

## Abstract

**Background:**

Severe acute respiratory syndrome coronavirus 2 (SARS-CoV-2) antibody testing in community settings may help us better understand the immune response to this virus and, therefore, help guide public health efforts.

**Aim:**

To conduct a seroprevalence study of immunoglobulin G (IgG) antibodies in Irish GP clinics.

**Design & setting:**

Participants were 172 staff and 799 patients from 15 general practices in the Midwest region of Ireland.

**Method:**

This seroprevalence study utilised two manufacturers’ point-of-care (POC) SARS-CoV-2 immunoglobulin M (IgM)—IgG combined antibody tests, which were offered to patients and staff in general practice from 15 June to 10 July 2020.

**Results:**

IgG seroprevalence was 12.6% in patients attending general practice and 11.1% in staff working in general practice, with administrative staff having the lowest seroprevalence at 2.5% and nursing staff having the highest at 17.6%. Previous symptoms suggestive of COVID-19 and history of a polymerase chain reaction (PCR) test were associated with higher seroprevalence. IgG antibodies were detected in approximately 80% of participants who had a previous PCR-confirmed infection. Average length of time between participants’ positive PCR test and positive IgG antibody test was 83 days.

**Conclusion:**

Patients and healthcare staff in general practice in Ireland had relatively high rates of IgG to SARS-CoV-2 compared with the national average between 15 June and 10 July 2020 (1.7%). Four-fifths of participants with a history of confirmed COVID-19 disease still had detectable antibodies an average of 12 weeks post-infection. While not proof of immunity, SARS-CoV-2 POC testing can be used to estimate IgG seroprevalence in general practice settings.

## How this fits in

The immune response to the SARS-CoV-2 virus continues to be characterised. This study examined for presence of IgG in 172 general practice staff and 799 patients from 15 June to 10 July 2020. While rates of seroprevalence were higher than estimated population-wide levels, they do not suggest COVID-19 has passed silently through our communities to any large degree. POC serologic studies can easily be integrated into routine general practice, and may help inform seroprevalence studies and other public health efforts required during this pandemic.

## Introduction

A novel virus, SARS-CoV-2, first detected in Wuhan, China, in December 2019,^1^ caused a global pandemic and >3.7 million deaths worldwide by 8 June 2021.^2^ The rapid spread of SARS-CoV-2, asymptomatic infection, and the requirement to understand how vaccination will impact the pandemic have fuelled interest in large-scale screening.^3–8^

The standard method for diagnosis of SARS-CoV-2 infection remains a nucleic acid real-time PCR test. Although these tests have low levels of false positives, they are limited by a relatively high rate of false-negative results owing to multiple influencing factors, including virus shedding rates and the technical difficulty of performing an effective nasopharyngeal swab.^9^ While rapid, easily deployed, POC testing has been employed successfully in cohorting patients with viral illness, specifically influenza,^10^ POC antigen tests to detect active SARS-CoV-2 infection in real-world settings, while evolving, have yet to replace PCR testing.^11,12^

While unlikely to impact on acute management, use of reliable POC tests for antibodies as a result of infection or vaccination may provide additional relevant information to public health authorities in their management of the pandemic.^5,8^ Many rapid, simple, POC lateral flow immunoassay (LFIA) tests to simultaneously detect IgM and IgG antibodies against SARS-CoV-2 in human blood within 15 minutes have been developed.^13,14^ Performance characteristics of these tests vary, although a majority have sensitivities of >80% when used after day 15 of infection.^15–17^ If this lag between infection and detectable antibody levels^17–19^ is accounted for, POC antibody tests can help identify who has been previously infected.^17,20^ How long such antibodies will persist is unclear, although studies thus far have confirmed their presence for at least 6 months.^21–24^

Much is still unknown about the immune response to COVID-19, such as whether antibodies created in response to infection or vaccination confer protection from severe disease, from reinfection, or for how long protection might be sustained.^23–28^ Reinfections, potentially by genomically different virus strains,^29,30^ are also a source of concern, as are new variant strains with increased transmissibility.^31^

Natural active immunity from previous infection seems to prevent reinfection in approximately 80% of cases (although this figure is lower for older patients),^29^ but the role IgG antibodies play is not well defined. It appears upwards of 80% of those infected by SARS-CoV-2 make IgG antibodies in response^25,26^ and this group continues to make IgG antibodies for >6 months,^23,27^ which is a figure that is being revised upwards as time passes. Titres do drop over time but numbers of memory B cells^27^ and the proportion of those with detectable antibodies seem to remain stable.^23^ However, the immune response is complex, involving memory B cells, antibodies, memory CD4+ T cells, and/or memory CD8+ T cells. Thus, it seems simple serological tests for SARS-CoV-2 antibodies may never adequately describe the 'richness and durability' of immune memory to COVID-19.^24^ Nevertheless, at population level, these tests may assist seroprevalence studies and assessment of those with long-term clinical sequelae.^28^

Therefore, well-designed studies, with clear reporting of time lag between diagnosed infections and antibody detection,^17^ to elucidate the mechanisms and serological correlates of protective immunity, may help guide rational clinical and public health policies.

As there is limited published information on the deployment of antibody POC tests in primary care settings, and none, to the authors’ knowledge, in the Irish population, the aim was to conduct a seroprevalence study among patients and healthcare workers in general practice in Ireland for SARS-CoV-2 using combined IgM–IgG antibody POC tests.

## Method

General practices of the University of Limerick Education and Research Network for General Practice (ULEARN-GP)^32^ were invited to participate by email and follow-up phone call. This network comprises 134 practices in Ireland, centred around the Midwest. Acquisition of 1000 POC testing kits from two manufacturers determined the number of practices that could be approached as it was intended to offer kits to practices in batches of 25–50. A selection of small and large practices from villages, towns, and cities in the Midwest were emailed an invitation to participate in the study. If an invited practice declined to participate, the next similar practice in the network was chosen until all POC tests were distributed. Details of participating practices are shown in Table 1.

**Table 1.** Characteristics of participating practices

Practice code	Practice location (village, town, or city)^a^	Practice size (small, medium, or large)^b^	Geographic spread (Clare, Limerick, or Tipperary)	Completed POC tests, *n*	Positive POC tests, either type positive, *n* (%)
1	Village	Small	Clare	97	6 (6)
2	Town	Medium	Clare	29	3 (10)
3	Town	Medium	Clare	49	4 (8)
4	Village	Small	Clare	50	0 (0)
5	Town	Large	Clare	52	1 (2)
6	Village	Small	Clare	50	6 (12)
7	Town	Medium	Limerick	322	74 (23)
8	Village	Small	Limerick	50	1 (2)
9	Village	Small	Limerick	17	2 (12)
10	City	Large	Limerick	50	4 (8)
11	City	Large	Limerick	50	2 (4)
12	City	Large	Limerick	50	2 (4)
13	Village	Medium	Limerick	45	10 (22)
14	Town	Medium	Tipperary	27	2 (7)
15	Town	Small	Tipperary	33	1 (3)

^a^Village (population <1500), town (population 1500–10 000), and city (population >10 000). ^b^Small (General Medical Services [GMS] list <1000), medium (GMS list 1000–2000), and large (GMS list >2000).

Participants needed to be aged ≥18 years and able to provide consent, and be either staff or a patient at one of the general practice sites. Exclusion criteria were: cognitive impairment; severe psychiatric illness; use of immunologic agents; or pregnancy. There were no incentives provided to these practices to participate, although personal protective equipment (PPE) was supplied along with the equipment required to perform the study.

Two different serological POC kits for qualitative analysis of IgG and IgM directed against the receptor-binding domain of the SARS-CoV-2 spike protein were used: SARS-CoV-2 IgM/IgG Antibody Assay Colloidal Gold Complex test (Maccura Biotechnology Ltd, Chengdu, China) and the 2019-nCoV IgG/IgM Antibody Rapid test (Diagreat Biotechnologies Co Ltd, Beijing, China) with reported sensitivity and specificity for IgG of 71% and 98%,^13^ and 83% and 93%,^33^ respectively. All testing was performed in GP settings over a 4-week period from 15 June to 10 July 2020, in accordance with the manufacturers’ directions. Of note, this study is not intended as an assessment of the diagnostic performance of the lateral flow assays used (see Supplementary Table S1).

For the study period, all patients attending participating practices for routine care were informed about the study taking place and that they could participate if they so wished. Patients were informed that they would be blinded to the results of their POC test. Interested patients were provided with an information leaflet, and allowed time to consider whether or not they wanted to participate. Those who consented to participate then chose whether to supply a pinprick capillary blood sample or a single ethylenediaminetetraacetic acid (EDTA) blood sample via venepuncture; several drops of blood were applied to a small test cartridge along with a buffer solution for each of two separate test kits, and results read 15 minutes later.

Finally, a brief clinical history was taken (see Supplementary Box S1) and recorded on a standardised data collection sheet in order to estimate onset of relevant symptoms since the beginning of the pandemic, if any, and previous PCR testing for SARS-CoV-2 via nasopharyngeal swab. Evidence of previous PCR results were obtained directly from patient testimony, although in practice, owing to the involvement of GPs in referral for community-based testing, many tests could be verified on the practice electronic medical record.

For quality assurance, 20 randomly selected samples from a bank of previously collected serum samples (10 samples from 2007 to 2008 and 10 samples from 2017 to 2018) were also tested with both kits as part of this study. All 20 samples were found to be negative, and the test control markers reacted appropriately and as specified.

Seroprevalence was defined as the proportion of participants who had a positive result for the IgG band on the two test kits used in the study. As in similar studies internationally, owing to the differences in sensitivity and specificity of the IgM antibody, its shorter duration, and the heterogeneity of IgM results seen previously,^18,19,34^ seroprevalence results presented herein are based only on IgG antibodies.

In order to convert the observed positive and negative values of each test to a population level estimate of seroprevalence, the reported data on sensitivity and specificity were used for each test.^13,33^ Of note, the probability an individual tests positive on a given test is the probability that they have true disease multiplied by the sensitivity of the test plus the probability that they do not have true disease multiplied by 1 — specificity of the test. Inverting this relationship allows estimation of true seroprevalence given the test results.

Since the estimated sensitivity and specificity of the tests are based on relatively small numbers,^13,33^ this process was carried out using Monte Carlo estimation and 95% confidence intervals (CIs) are reported. The Strengthening the Reporting of Observational Studies in Epidemiology (STROBE) checklist^35^ was followed during design of this study. Analyses were completed using R (version 4.0.5) and IBM SPSS Statistics (version 24.0).

## Results

### Practice recruitment

Fifteen practices were approached to participate in the study and all agreed to do so, with allocation of POC tests varying according to practice size and scope of practices to recruit during the 4-week study period (Table 1).

### Participant recruitment and characteristics

From these practices, 971 participants (*n* = 799 patients, *n* = 172 staff) were recruited and tested using both POC test kits (Table 2). Venepuncture was favoured by 910 participants. The number of participants drawn from each practice was determined by the size of the practice and whatever was feasible for the practice to recruit in the 4 weeks of the study. The mean age of participants was 53.0 years (standard deviation [SD] 17.2) with an age range of 18–97 years. Of the entire cohort, 60% were female, 82% were patients, and the remainder (18%) were healthcare staff with similar numbers of administration staff, nursing staff, doctors, and other healthcare staff across this group. With regard to previous testing, 139 (14%) had undergone prior PCR testing for SARS-CoV-2 and 33 of these (24%) had tested positive.

**Table 2.** Baseline characteristics of all participants, including previous symptoms, PCR testing, and antibody detection

**Participant characteristics**	**Total, *n* = 971**	**Previous symptoms suggestive of SARS-CoV-2,** ***n* = 339**	**Previous PCR nasopharyngeal swab for SARS-CoV-2,** ***n* = 139**	**Either antibody test positive,** ***n* = 118**
**Age, years**	**Mean**	**SD**	**Mean**	**SD**	**Mean**	**SD**	**Mean**	**SD**
	53.0	17.2	49.8	15.4	51.7	13.6	50.3	15.8
	**Median**	**Range**	**Median**	**Range**	**Median**	**Range**	**Median**	**Range**
	55	18–97	51	18–88	53	20–84	54	18–80
**Sex**	**Patients, *n***	**Patients, %**	**Patients, *n***	**Patients, %**	**Patients, *n***	**Patients, %**	**Patients, *n***	**Patients, %**
Male	392	40	120	35	50	36	46	39
Female	579	60	219	65	89	64	72	61
**Participant type**								
1. Patient	799	82	271	80	93	67	98	83
2. Healthcare staff	172	18	68	20	46	33	20	17
2.1 Administration	34	4	10	3	6	4	2	2
2.2 Nursing	51	5	24	7	16	12	8	7
2.3 Doctor	40	4	14	4	14	10	4	3
2.4 Other healthcare staff	47	5	20	6	10	7	6	5
**History PCR and symptoms**								
Previous PCR test for SARS-CoV-2	139	14	108	32	139	100	43	36
Positive PCR test	33	24	28	26	33	24	27	63
Previous symptoms suggestive of SARS-CoV-2	339	35	—	—	108	78	83	70
**Severity of symptoms**								
Mild	112	12	112	33	22	16	23	19
Moderate	193	20	193	57	70	50	52	44
Severe	34	4	34	10	16	12	8	7

PCR = polymerase chain reaction. SD = standard deviation.

Regarding previous symptoms suggestive of SARS-CoV-2, 339 (35%) of all participants described such symptoms, of which the majority (57%) were 'moderate' in severity. Less than one-third (32%) of those patients with previous relevant symptoms had undergone a PCR test for SARS-CoV-2, and 17% of those who were seropositive were asymptomatic in the months preceding the testing.

### Seroprevalence

Of the 971 participants, 967 had valid test results using the Maccura test kit and 918 using the Diagreat test kits, with 914 participants having valid test results for both test kits (see Table 3 for full results for both kits). One practice only used a single test kit (Maccura) instead of both on all participants resulting in reduced numbers for the Diagreat test. A control strip did not appear for a single participant for each test kit (99.9% performance for the control strip for each test kit) so both of these results were excluded from the final analysis.

**Table 3.** Overall seroprevalence of IgG antibodies to SARS-CoV-2 on point-of-care testing by participant characteristics

	**Maccura test negative, valid participants, *n***	**Maccura test positive, valid participants, *n***	**Diagreat test negative, valid participants, *n***	**Diagreat test positive, valid participants, *n***	**Seroprevalence estimate via Maccura, %**	**Seroprevalence estimate via Maccura, 95% CI**	**Seroprevalence estimate via Diagreat, %**	**Seroprevalence estimate via Diagreat, 95% CI**
Overall	868	99	812	106	12.3	7.4 to 16.0	4.5	0.0 to 14.7
**Sex**								
Female	518	59	484	62	12.3	7.1 to 16.8	4.9	0.0 to 15.9
Male	350	40	328	44	12.3	6.6 to 17.7	4.3	0.0 to 14.8
**Age, years**								
18–34	143	22	118	26	16.8	9.1 to 25.6	1.3	0.0 to 12.8
35–49	212	20	199	20	9.9	3.8 to 16.4	6.0	0.0 to 17.1
50–64	256	43	247	42	18.3	11.8 to 25.0	8.5	0.0 to 20.3
≥65	257	14	248	18	5.0	0.0 to 10.0	0.0	0.0 to 9.2
**Participant type**								
Patient	713	83	674	88	12.6	7.5 to 16.6	4.5	0.0 to 14.8
Healthcare worker	155	16	138	18	11.1	4.2 to 18.6	4.5	0.0 to 17.3
Administration	32	1	31	1	2.5	0.0 to 16.7	0.0	0.0 to 10.6
Nursing	44	7	41	8	17.6	5.9 to 33.8	10.7	0.0 to 30.6
Doctor	36	4	35	4	12.3	1.5 to 29.6	3.0	0.0 to 22.3
Other healthcare staff	43	4	31	5	10.1	0.6 to 24.9	7.7	0.0 to 29.2
**History PCR and symptoms**								
Previous symptoms suggestive of SARS-CoV-2	266^a^	73	257	77	28.7	21.7 to 36.0	19.9	0.0 to 32.8
No previous symptoms suggestive of SARS-CoV-2	601^a^	26	555	29	3.5	0.0 to 6.9	0.0	0.0 to 5.7
Previous PCR nasopharyngeal swab for SARS-CoV-2	99	39	97	38	38.5	27.7 to 50.6	26.6	0.0 to 43.4
No previous PCR nasopharyngeal swab for SARS-CoV-2	769	60	715	68	8.0	3.1 to 11.5	0.6	0.0 to 10.8

^a^One patient did not have a valid option recorded. PCR = polymerase chain reaction.

Seroprevalence was estimated with greater precision by the Maccura test owing to the greater amount of performance data available^13^ on its sensitivity and specificity. Seroprevalence based on the Maccura test was estimated to be 12.6% (95% CI = 7.5% to 16.6%) in patients attending general practice and 11.1% (95% CI = 4.2% to 18.6%) in staff working in general practice. Administrative staff exhibited lowest seroprevalence at 2.5% (95% CI = 0.0% to 16.7%) and nursing staff the highest at 17.6% (95% CI = 5.9% to 33.8%). Seroprevalence was similar in males and females; was lowest in those aged ≥65 years; and increased in those who had previous symptoms suggestive of SARS-CoV-2 or who had undergone a PCR test for SARS-CoV-2.

Of the 339 (35%) participants who reported symptoms since the beginning of the pandemic, 83 (24% of those reporting symptoms) demonstrated evidence of IgG to SARS-CoV-2.

For the 33 patients with previous PCR-confirmed COVID-19 infection, 27 (82% of previously infected individuals) showed serologic evidence of IgG to SARS-CoV-2 with one or both of the kits. For this subgroup, mean duration between their positive PCR test and the positive serology test for IgG was 83 days (95% CI = 77 to 89). Of note, 28 of the 33 PCR-positive patients reported symptoms of COVID-19 before previously testing positive for COVID-19, whereas the remaining five were asymptomatic prior to their positive PCR test..

For the six previously diagnosed patients who did not show evidence of IgG on either test kit, mean duration between their positive PCR test and the serology test was 77 days (95% CI = 44 to 111). Five of these patients reported symptoms of COVID-19 before their positive PCR test.

### Relative performance of POC tests with PCR testing

Of the participants who had a valid Maccura test, 33 had previously tested positive on PCR for SARS-CoV-2 and 26 tested positive for IgG, demonstrating 79% (95% CI = 64% to 94%) positivity. For the Diagreat test, 32 had previously tested positive on PCR for SARS-CoV-2 and 25 tested positive for IgG on the Diagreat test, demonstrating 78% (95% CI = 63% to 93%) positivity (Table 4).

**Table 4.** Relationship between Diagreat and Maccura IgG test results and previously positive PCR tests for SARS-CoV-2

	**IgG negative, *n***	**IgG positive, *n***	**Total, *n***
**Diagreat Test**			
PCR negative	89	13	102
PCR positive	7	25	32
Total	96	38	134
**Maccura Test**			
PCR negative	92	13	105
PCR positive	7	26	33
Total	99	39	138

IgG = immunoglobulin G. IgM = immunoglobulin M. PCR = polymerase chain reaction.

## Discussion

### Summary

Seroprevalence for SARS-CoV-2 IgG antibodies was 12.6% for patients attending general practice and 11.1% for practice staff. Nursing staff demonstrated the highest seroprevalence levels while administrative staff, presumably owing to relatively less direct patient contact, had lowest seroprevalence. Seroprevalence was similar across sex, was lowest in those aged ≥65 years, and was increased in those who had previous symptoms suggestive of COVID-19 or who had undergone a PCR test for COVID-19.

Notably, more than two-thirds of participants with previous symptoms suggestive of COVID-19 had not undergone a PCR test for COVID-19. This likely reflects the fact that there was limited availability of PCR tests, and thus more stringent criteria for testing in Ireland in the early stages of the pandemic.^36^

Results across practices varied from no detection to IgG detection in 23% of participants, presumably owing to considerable variability in COVID-19 geographic spread early in the pandemic. The vast majority of participants chose venepuncture to provide the blood sample for analysis, presumably owing to the fact that obtaining several drops of blood by capillary blood sampling would be more painful. Additionally, many of these patients were likely to be attending general practice that day for phlebotomy, and participation in the study did not mean any additional venepuncture.

While recognising not all those with PCR-confirmed COVID-19 infection will have produced IgG in response^25,26^ and IgG levels can wane,^27^ IgG antibodies were detected in 82% of participants with a confirmed prior COVID-19 infection, with a mean duration of nearly 3 months between diagnosis of infection and antibody detection.

### Strengths and limitations

Key strengths of this study were the large number of patients from a reasonably wide selection of practices, which were recruited to the study with good representation across age and sex. Limitations include restriction of the study to the Midwest of Ireland and limited numbers of healthcare staff, meaning seroprevalence rates of the various staff categories had wide CIs. However, practices were purposefully selected to represent the entire region and different communities within the region, which is borne out by the differing seroprevalence rates recorded.

Patients and staff who had a history of COVID-19 infection or suggestive symptoms may have been more willing to participate in the study to assess their 'immunity' and thus further studies are needed to establish true prevalence of antibodies in GP staff and patients, and how this is changing over time.

An additional limitation arises from using a previous positive PCR result as a proxy for true SARS-CoV-2 infection in the seroprevalence analyses, as PCR tests have their own fallibilities. Similarly, any POC testing kit has inherent limitations, and IgG levels to SARS-CoV-2 are also known to drop in some patients over time.^23,27^ However, even with these limitations, the analyses reveal plausible seroconversion rates that help describe the known previous clinical history of many people in the study.

Finally, some individuals do not develop a detectable antibody response for a variety of reasons, such as T-cell immune response, and these prior infections will not be detected in a seroprevalence study.^24,28,37,38^

### Comparison with existing literature

Healthcare workers have been at higher risk of COVID-19 infection than the general population owing to exposure to infected patients and crowded enclosed environments.^39–41^ They have been shown to be more than 10 times more likely to contract COVID-19 compared with the general public,^31^ and evidence suggests hospital staff without direct patient contact are also at higher risk of infection.^32^

From the patient perspective, those with chronic conditions spend more time in general practice and other healthcare settings as they often need closer monitoring and more frequent treatment.^42^ It is plausible that such patients with increased medical need continued to visit many healthcare settings during the pandemic, thereby increasing their risk of contracting COVID-19 within these high-risk environments.

At the same time as the study was carried out, a national seroprevalence study (SCOPI)^43^ was undertaken by the Irish Health Protection Surveillance Centre (HPSC). This involved cross-sectional sampling of people living in two areas in Ireland: one with a high incidence of confirmed COVID-19 cases (Dublin); and the other with a lower incidence of confirmed COVID-19 cases (Sligo). The findings of the study were then extrapolated to estimate an overall prevalence for Ireland of 1.7%,^43^ giving the participants in the present study a rate of infection more than seven times the national average at the time.

More recently, a large study of 5700 healthcare workers in two hospitals situated in areas of high COVID-19 incidence (Dublin) and lower incidence (Galway) has shown prevalence of SARS-CoV-2 IgG antibodies in 15.0% and 4.1% of participants, respectively.^44^

To the authors’ knowledge, there are no seroprevalence data from community healthcare settings in Ireland available, and indeed little internationally, but data are available from Spain where a seroprevalence rate of 5.5%–5.9% has been demonstrated.^45,46^ The present study makes a valuable contribution to the knowledge regarding potential levels of circulating SARS-CoV-2 in such settings in Ireland and the methodology may assist future similar studies in Irish general practice.

As studies with longer follow-up emerge,^21–28^ persistence of antibodies in a majority of infected individuals for a reasonable length of time may be welcome news for vaccination efforts and indeed for those patients who have overcome COVID-19 infection. However, what IgG levels mean for protection from subsequent infection remains to be seen.

### Implications for research and practice

Patients and healthcare staff in general practice in Ireland demonstrated higher evidence of COVID-19 infection than seen in the general public nationally and similar to rates seen in hospital-based healthcare workers in areas of high-disease activity.

Venepuncture was chosen over capillary blood sampling by most participants and routine venepuncture for other reasons in general practice may provide opportunities for future seroprevalence studies.

The number of individuals with detectable levels of IgG, if taken as a proxy for some degree of immunity, while greater than the national average, was far below that of a reasonable population-level 'herd immunity' threshold.^38^ This implies COVID-19 has not swept silently through community settings, and highlights the importance of continued compliance with simple public health measures until vaccination efforts mature and more is learnt about their effect on this pandemic.

Comparison of positive PCR results and the POC test kits demonstrates how use of distinct testing methods on the same participants can offer some quality assurance and compensation for each test’s inherent fallibilities and differing sensitivities. While POC tests used in the study may not be conclusive about immunity to any or all strains of SARS-CoV-2, it seems prudent to continue study of this important aspect of the immune system’s response to this novel virus.
